# Supplementary material for: Molecular composition of organic matter controls methylmercury formation in boreal lakes
Source: Nat Commun. 2017 Feb 9;8:14255. doi: 10.1038/ncomms14255 (PMC5309796; doi:10.1038/ncomms14255)
Supplement: Supplementary Information — Supplementary Figures and Supplementary Tables [file ncomms14255-s1.pdf]

Supplementary Table 1. Characteristics of the investigated lakes and chemical properties of the water overlying sediment. Sampled depth in the profundal zone is equal to the maximum lake depth (Zmax). Lake codes: Lilla Sångaren (LS), Ljustjärn (LJU), Svarttjärn (S), Fälaren (F), Oppsveten (O), Strandsjön (STR), Valloxen (V), Vallentunasjön (VALE), Marnästjärn (M) and Lötsjön (LOTS).

	z	pH	T	C	O_2	DOC	TP	Chla	SO_4^{2-}	$SUVA_{254}$	'S 275-295'	'S ratio'
**Lake**	m		°C	µS s^{-1}	mg L^{-1}	mg L^{-1}	µg L^{-1}	µg L^{-1}	mg L^{-1}	L mg^{-1} m^{-1}	nm^{-1}	
LS	17.0	6.9	5	60	4.65	7.0	23	1.8	2.9	4.21	0.012	0.694
LJU	10	7.3	6.9	77	0.22	6.5	96	93	2.2	1.6	0.010	0.666
S	6.5	5.6	4.8	59	0.08	22.0	36	2.7	0.9	5.96	0.011	0.702
F	2.0	7.5	18.7	67	8.56	32.6	20	8.9	3.0	3.9	0.015	0.881
O	10.0	6.3	8.6	30	0.79	16.7	14	udl	2.3	4.78	0.012	0.705
STR	2.5	6.9	16.4	285	0.34	19.6	60	13.1	4.6	3.0	0.017	0.916
V	6.0	8.5	18.8	502	0.14	12.3	49	52	8.9	2.5	0.020	1.107
VALE	4	7.1	17.2	469	0.24	14.0	77	58	16.2	1.4	0.021	1.002
M	2.0	7.2	17.8	185	6.30	9.2	185	190	3.6	1.8	0.019	0.955
LOTS	7	6.8	11.5	288	0.3	13.3	65	18	1.60	1.7	0.019	0.896

Supplementary Table 2. Inorganic Hg and MeHg concentrations, Hg methylation (k_m) and MeHg demethylation rates (k_d), and characteristics of the investigated lakes: Lilla Sångaren (LS), Ljustjärn (LJU), Svarttjärn (S), Fälaren (F), Oppsveten (O), Strandsjön (STR), Valloxen (V), Vallentunasjön (VALE), Marnästjärn (M) and Lötsjön (LOTS).

Sediment characteristics											
**Lake**	IHg ng g^{-1}	MeHg ng g^{-1}	MeHg %	k_m day^{-1}	k_d day^{-1}	k_m/k_d	TC %	TN %	TP %	C/N	BP µgC L^{-1} d^{-1}
LS 0-1	209±4	7.2±0.2	3	0.006 ± 0.000	0.033 ± 0.000	0.183	20	1.34	0.020	14.78	4.32
LS 1-2	253±2	5.9±0.04	2	0.013 ± 0.001	0.030 ± 0.001	0.432	18	1.20	0.017	15.34	3.70
LJU 0-1	255±26	3.8±0.5	1.5	0.009 ± 0.002	0.127 ± 0.005	0.071	32	2.48	0.012	12.81	0.40
LJU 1-2	267±49	4.2±0.2	1.5	0.010 ± 0.002	0.077 ± 0.001	0.130	32	2.45	0.011	13.08	0.48
S 0-1	380±14	8±0.4	2	0.011 ± 0.001	0.014 ± 0.000	0.767	24	1.54	0.011	15.77	2.63
S 1-2	365±35	6.4±2	2	0.011 ± 0.001	0.101 ± 0.022	0.109	23	1.43	0.011	16.15	2.69
F 0-1	238±2	4±0.1	1.7	0.007 ± 0.000	0.086 ± 0.001	0.081	25	1.96	0.010	12.55	7.69
F 1-2	231±17	3.7±0.3	1.6	0.017 ± 0.002	0.058 ± 0.000	0.293	24	1.94	0.011	12.45	5.04
O 0-1	218±4	6.1±1.3	3	0.011 ± 0.001	0.099 ± 0.005	0.111	19	1.10	0.012	17.29	1.66
O 1-2	287±47	6.6±1.4	2	0.014 ± 0.004	0.104 ± 0.027	0.135	19	1.10	0.014	17.66	2.18
STR 0-1	178±14	1.4±0.6	0.8	0.015 ± 0.003	0.298 ± 0.009	0.050	12	1.35	0.015	8.64	16.74
STR 1-2	180±8	1.3±0.6	0.7	0.011 ± 0.002	0.265 ± 0.021	0.041	12	1.34	0.014	8.81	16.39
V 0-1	74±2	2.8±0.5	3.6	0.077 ± 0.000	0.118 ± 0.000	0.653	14	1.72	0.015	8.16	21.16
V 1-2	73±2	2.1±0.2	2.9	0.078 ± 0.001	0.109 ± 0.001	0.716	14	1.71	0.015	8.02	26.01
VALE 0-1	107±15	1.9±0.6	1.8	0.066 ± 0.007	0.254 ± 0.005	0.260	19	2.37	0.018	7.89	22.91
VALE 1-2	96±6	2.1±0.4	2.1	0.052 ± 0.003	0.219 ± 0.008	0.237	19	2.38	0.018	8.00	19.67
M 0-1	12599±211	96±18	0.8	0.047 ± 0.001	0.115 ± 0.004	0.409	20	2.21	0.025	9.04	13.22
M 1-2	12822±162	104±3	0.8	0.030 ± 0.000	0.123 ± 0.009	0.244	19	2.14	0.024	9.10	15.94
LOTS 0-1	150±14	2.4±0.3	1.6	0.077 ± 0.007	0.192 ± 0.034	0.401	14	1.75	0.021	7.95	15.57
LOTS 1-2	162±3	2.8±0.9	1.7	0.079 ± 0.002	0.198 ± 0.053	0.399	15	1.74	0.021	8.48	18.79

Supplementary Table 3. Relative abundances of different groups of organic compounds in the sediments of the ten studied lakes (average of two depths). The values are given as follows: average ± standard deviation considering the two sediment depths, i.e. 0-1 and 1-2 cm. The light grey background denotes average values below 10 % of the all-lake average; ii) no background denotes values close to the all-lake average; iii) dark grey background represents values above 10 % of the all-lake average.

		LS	LJU	S	F	O	STR	V	VALE	M	LOTS
High molecular weight carbohydrates	Plant/fresh	1.6 ± 1.2	9.4 ± 0.1	10 ± 2	4.0 ± 0.2	0.7 ± 0.4	0.8 ± 0.2	2.5 ± 1.7	13 ± 2	7.9 ± 0.3	5.1 ± 0.3
Furans	Plant/Degraded	12.9 ± 0.1	7.4 ± 0.1	10.8 ± 0.3	7.7 ± 0.1	13 ± 1	11.4 ± 0.4	5.4 ± 0.1	5.8 ± 0.1	5.2 ± 0.1	8.1 ± 0.2
Total carbohydrates		25.5 ± 0.2	31.4 ± 0.2	34. ± 2	28.4 ± 0.4	25.3 ± 0.9	24.9 ± 0.2	18 ± 2	32 ± 2	24.7 ± 0.5	25.3 ± 0.6
Phenols	Plant	6.5 ± 0.4	5.81 ± 0.01	7.81 ± 0.01	6.7 ± 0.2	7.2 ± 0.8	6.7 ± 0.5	4.0 ± 0.1	3.5 ± 0.1	5.2 ± 0.1	5.23 ± 0.03
Lignin	Plant	3.2 ± 0.2	5.6 ± 0.2	3.6 ± 0.3	7.4 ± 0.2	3.1 ± 0.5	4.1 ± 0.1	2.57 ± 0.03	1.30 ± 0.03	4.1 ± 0.1	4.09 ± 0.02
n-alkanes C27-29	Plant	3.0 ± 0.1	4.56 ± 0.01	3.8 ± 0.3	3.65 ± 0.03	2.6 ± 0.8	2.28 ± 0.01	2.36 ± 0.02	2.75 ± 0.02	3.9 ± 0.1	3.1 ± 0.1
Alkan-2-ones C29-33	Plant	0.90 ± 0.01	2.05 ± 0.01	1.15 ± 0.06	1.12 ± 0.08	0.9 ± 0.4	0.72 ± 0.01	0.81 ± 0.01	0.75 ± 0.04	0.95 ± 0.03	1.7 ± 0.2
Proteins	Algae	2.3 ± 0.2	3.0 ± 0.1	3.3 ± 0.1	3.23 ± 0.03	2.00 ± 0.03	4.3 ± 0.1	5.4 ± 0.2	5.8 ± 0.1	5.0 ± 0.2	4.7 ± 0.1
Pyrrole + pyridine	Algae/Degraded	5.0 ± 0.1	2.9 ± 0.1	3.3 ± 0.1	3.93 ± 0.01	5.7 ± 0.8	5.7 ± 0.2	3.38 ± 0.01	2.8 ± 0.1	3.04 ± 0.03	4.2 ± 0.2
Aromatic nitriles	Algae/Degraded	1.33 ± 0.01	0.39 ± 0.01	0.49 ± 0.06	0.71 ± 0.02	1.3 ± 0.5	1.26 ± 0.03	0.86 ± 0.02	0.67 ± 0.01	0.70 ± 0.04	0.90 ± 0.02
Chitin-compounds	Micro-organisms	2.4 ± 0.3	2.29 ± 0.04	1.7 ± 0.2	2.8 ± 0.1	2.3 ± 0.1	3.8 ± 0.1	2.10 ± 0.01	2.10 ± 0.01	2.5 ± 0.1	3.0 ± 0.1
Total N compounds		14.3 ± 0.4	10.8 ± 0.4	11.8 ± 0.1	14.0 ± 0.2	14 ± 2	20.8 ± 0.1	17.7 ± 0.5	17.0 ± 0.2	16.8 ± 0.3	17.8 ± 0.2
Chlorophyll	Algae/fresh	1.36 ± 0.03	1.7 ± 0.1	1.78 ± 0.06	1.41 ± 0.02	0.67 ± 0.06	1.1 ± 0.1	7.2 ± 0.1	4.9 ± 0.4	4.9 ± 0.2	3.2 ± 0.3
n-alkanes C17-20	Algae	2.7 ± 0.1	2.3 ± 0.1	1.9 ± 0.1	2.64 ± 0.01	3.1 ± 0.2	3.2 ± 0.2	1.7 ± 0.2	2.25 ± 0.04	1.9 ± 0.1	2.4 ± 0.1
n-alkenes C17-20	Algae	1.96 ± 0.01	2.20 ± 0.05	2.2 ± 0.1	2.8 ± 0.2	1.6 ± 0.2	2.8 ± 0.4	21 ± 1	13 ± 1.	8.9 ± 0.4	5.8 ± 0.4
n-alkanes C11-13	Degraded	1.9 ± 0.1	1.26 ± 0.01	1.1 ± 0.1	1.6 ± 0.2	2.15 ± 0.02	1.8 ± 0.1	1.1 ± 0.1	1.08 ± 0.03	1.03 ± 0.03	1.3 ± 0.1
n-alkenes C11-13	Degraded	1.95 ± 0.03	1.39 ± 0.01	1.10 ± 0.08	2.24 ± 0.01	1.88 ± 0.02	2.1 ± 0.1	1.58 ± 0.03	1.8 ± 0.1	1.70 ± 0.02	1.7 ± 0.1
n-alkanes C22-24	Resistant	0.77 ± 0.01	1.01 ± 0.02	0.67 ± 0.04	1.02 ± 0.02	0.9 ± 0.1	0.95 ± 0.01	0.46 ± 0.07	0.64 ± 0.02	0.69 ± 0.02	0.77 ± 0.03
n-alkenes C22-24	Resistant	1.7 ± 0.2	1.77 ± 0.01	1.5 ± 0.3	1.8 ± 0.1	2.5 ± 0.7	1.9 ± 0.2	0.73 ± 0.04	0.98 ± 0.04	1.04 ± 0.01	1.6 ± 0.1
Sterols	Fresh	1.0 ± 0.3	2.5 ± 0.3	2.34 ± 0.7	0.82 ± 0.03	0.4 ± 0.2	0.09 ± 0.07	0.6 ± 0.1	0.70 ± 0.01	2.31 ± 0.03	0.26 ± 0.07
Sterenes-sterenones	Degraded	8.9 ± 0.8	7.2 ± 0.2	5.8 ± 0.5	3.0 ± 0.2	6.1 ± 0.7	2.23 ± 0.05	2.55 ± 0.08	2.03 ± 0.02	2.98 ± 0.09	5.44 ± 0.06
(Poly)aromatics	Degraded	7.7 ± 0.2	4.4 ± 0.2	5.3 ± 0.3	5.5 ± 0.2	8 ± 1	8.2 ± 0.4	7.2 ± 0.2	6.3 ± 0.6	7.5 ± 0.1	7 ± 1
N compounds : carbohydrates	Algae : plant	0.56 ± 0.02	0.35 ± 0.02	0.35 ± 0.02	0.49 ± 0.01	0.56 ± 0.05	0.84 ± 0.01	0.98 ± 0.07	0.53 ± 0.03	0.68 ± 0.01	0.7 ± 0.01
Chlorophyll : (lignins + long-chain alkanes & alkan-2-ones)	Algae : plant	0.19 ± 0.01	0.14 ± 0.01	0.21 ± 0.02	0.12 ± 0.01	0.10 ± 0.02	0.15 ± 0.02	1.25 ± 0.04	1.02 ± 0.08	0.55 ± 0.02	0.36 ± 0.04

Supplementary Table 4. Differences in TC and TN contents as well as in the relative abundances of proteins, levosugars (fresh carbohydrates) and phytol (fresh chlorophyll) between the sediments at 0-1 and 1-2 cm. Decreases on the relative abundance of specific organic compounds with depth are highlighted in orange. Decreases in the relative abundance of the organic compounds that fall into 5-10% (analytical and/or sampling uncertainty) are highlighted in green. Increases in the relative abundance of these labile organic compounds with sediment depth for some lakes are highlighted in blue.

	**TC (%)**		**TN (%)**		**Levosugar**		**Proteins**		**Phytol**	
	Individual values	**Av** ± sd *(rsd)*	Individual values	**Av** ± sd *(rsd)*	Individual values	**Av** ± sd *(rsd)*	Individual values	**Av** ± sd *(rsd)*	Individual values	**Av** ± sd *(rsd)*
F 0-1<br>F 1-2	24.6<br>24.2	**24.4** ± 0.3<br>*(1%)*	1.96<br>1.94	**1.95** ± 0.01<br>*(1 %)*	4.0<br>3.8	**3.9** ± 0.1<br>*(4%)*	3.25<br>3.20	**3.23**± 0.03<br>*(1%)*	0.21<br>0.16	**0.18**± 0.04<br>*(21%)*
LJU 0-1<br>LJU 1-2	31.77<br>32.04	**31.9** ± 0.2<br>*(1%)*	2.48<br>2.45	**2.47** ± 0.02<br>*(1 %)*	8.5<br>8.6	**8.54** ± 0.05<br>*(1%)*	3.02<br>2.91	**2.96**± 0.08<br>*(6%)*	0.11<br>0.09	**0.10**± 0.01<br>*(12%)*
LOTS0-1<br>LOTS 1-2	13.91<br>14.76	**14.3** ± 0.6<br>*(4%)*	1.75<br>1.74	**1.75** ± 0.01<br>*(0.4 %)*	5.1<br>4.7	**4.9** ± 0.3<br>*(8%)*	4.83<br>4.64	**4.7**± 0.1<br>*(3%)*	0.95<br>0.83	**0.89**± 0.09<br>*(10%)*
LS 0-1<br>LS 0-2	19.8<br>18.4	**19 ± 1**<br>*(5%)*	1.34<br>1.20	**1.3** ± 0.1<br>*(8 %)*	0.6<br>2.3	**1.4** ± 1.2<br>*(86%)*	2.41<br>2.18	**2.3**± 0.2<br>*(7%)*	0.09<br>0.08	**0.8** ± 0.1<br>*(12%)*
M 0-1<br>M 1-2	19.97<br>19.48	**19.7** ± 0.3<br>*(2%)*	2.21<br>2.14	**2.18**± 0.05<br>*(2 %)*	7.5<br>7.3	**7.4** ± 0.1<br>*(2%)*	5.11<br>4.89	**5.0**± 0.2<br>*(3%)*	2.05<br>2.22	**2.1**± 0.1 *(6%)*
O 0-1<br>O 1-2	19.0<br>19.4	**19.2** ± 0.3<br>*(2%)*	1.10<br>1.10	**1.10**± 0.01<br>*(1 %)*	0.4<br>0.9	**0.6** ± 0.4<br>*(56%)*	2.02<br>1.97	**1.99**± 0.03<br>*(1%)*	0.04<br>0.06	**0.05**± 0.01<br>*(22%)*
S 0-1<br>S 1-2	24.3<br>23.1	**23.7** ± 0.8<br>*(4 %)*	1.54<br>1.43	**1.49**± 0.08<br>*(5 %)*	11.2<br>8.2	**10** ± 2<br>*(26%)*	3.35<br>3.19	**3.3**± 0.1<br>*(3%)*	0.09<br>0.08	**0.08**± 0.01<br>*(10%)*
STR 0-1<br>STR 1-2	11.66<br>11.81	**11.7** ± 0.1<br>*(1 %)*	1.35<br>1.34	**1.35**± 0.01<br>*(0.05 %)*	1.0<br>0.6	**0.8** ± 0.2<br>*(31%)*	4.34<br>4.26	**4.3**± 0.06<br>*(2%)*	0.05<br>0.08	**0.07**± 0.02<br>*(31%)*
V 0-1<br>V 1-2	**14.0**<br>**13.7**	**13.9** ± 0.2<br>*(2 %)*	**1.72**<br>**1.71**	**1.72**± 0.01<br>*(0.4 %)*	3.6<br>1.2	**2.4** ± 1.7<br>*(72%)*	5.58<br>5.25	**5.4**± 0.2<br>*(5%)*	**4.61**<br>**4.97**	**4.8**± 0.3 *(5%)*
VALE 0-1<br>VALE 1-2	18.69<br>19.04	**18.9** ± 0.2<br>*(1 %)*	2.37<br>2.38	**2.38**± 0.03<br>*(0.3 %)*	13.7<br>11.6	**13** ± 2<br>*(12%)*	5.84<br>5.83	**5.84** ± 0.01<br>*(0.2%)*	2.49<br>3.04	**2.8**± 0.4<br>*(14%)*

Supplementary Table 5. Identified pyrolytic organic compounds along with their molecular mass (M), specific mass fragments of their MS spectra and information about their degradation status (D: degraded, F: fresh and U: unknown) and sources (U: unknown or ubiquitous, P: plant, A: algae, I: invertebrates and B: bacteria) according to the review table reported in Tolu *et al.* (2015)*.

Name	Formula	M	Specific mass fragments	Ref		
**Carbohydrates**						
3(2H)-furanone	$C_4H_4O_2$	84	**54**+55+**84**	[1]	D	U
3-furaldehyde	$C_5H_4O_2$	96	39+67+**95**+96	NIST	D	U
2-furaldehyde	$C_5H_4O_2$	96	39+67+**95**+96	NIST	D	U
Furanmethanol	$C_5H_6O_2$	98	41+53+69+81+**98**	NIST	U	U
4-Cyclopentene-1,3-dione	$C_5H_4O_2$	96	42+54+68+**96**	NIST	D	U
2-acetyl-furan	$C_6H_6O_2$	110	39+**95**+110	NIST	D	U
Methyl-2-furaldehyde	$C_6H_6O_2$	110	43+109+**110**	NIST	D	U
Dihydro-methyl-furanone	C_5H_6O	98	42+55+69+**98**	[1]	U	U
Methyl-2-furaldehyde	$C_6H_6O_2$	110	43+109+**110**	NIST	D	U
4-hydroxy-5,6-dihydro-pyran-2-one	$C_5H_6O_3$	114	42+43+57+**58**+85+**114**	[1]	U	U
2-hydroxy-1-methyl-1-Cyclopenten-3-one	$C_6H_8O_2$	112	41+55+69+84+**112**	NIST	D	U
Dianhydro-rhamnose	$C_6H_8O_3$	128	58+113+128	[2]	F	U
Anhydropentose	unknown		**57+73**	[2]		U
2,5-Dimethyl-4-hydroxy-furanone	$C_6H_8O_3$	128	**43**+57+85+**128**	NIST	U	U
Maltol	$C_6H_6O_3$	126	43+55+71+97+**126**	NIST	F	U
Levosugars (Levogalactosan)	unknown	162	57+**60**+70+**73**+98	[1]	F	P
1,4:3,6-Dianhydro-α-d-glucopyranose	$C_6H_6O_4$	144	57+69+85+98+144	[1]	F	U
2,3 dihydro-benzofuran	C_8H_8O	120	**91**+92+119+**120**	NIST	D	U
Anhydrohexose	unknown		**60**+71+**73**	[1]	F	U
Levosugars(Levomannosan)	unknown	162	57+**60**+70+**73**+98	[1]	F	P
Levosugars (Levoglucosan)	unknown	162	57+**60**+70+**73**+98	[1]	F	P
**Chitin derived compounds**						
Acetamide	C_2H_5NO	59	42+43+**44+59**	NIST	U	I
3-acetamido-furan	$C_6H_7NO_2$	125	43+53+54+**83**+125	NIST	U	I
3 acetamido-4-pyrone	unknown	153	55+68+83+**111**+153	[3]	U	I
Oxazoline structure	unknown		55+83+84	[3]	U	I
**N compounds**						
Pyridine + pyrrole	C_5H_5N	79	39+50+51+52+**79**	NIST	D	U
Maleimide	$C_4H_3NO_2$	97	54+69+**97**	NIST	U	U
Succinimide	$C_4H_5NO_2$	99	59+99	NIST	U	U
Benzeneacetonitrile	C_8H_7N	117	77+89+90+116+**117**	NIST	D	U
Benzenepropanenitrile	C_9H_9N	131	91+131	NIST	D	U
Picolinamide	$C_6H_6N_2O$	122	52+**79**+122	NIST	F	U
Indole	C_8H_7N	117	39+63+90+91+**117**	NIST	U	U
Methyl-indole	C_9H_9N	131	77+103+**130+131**	NIST	U	U
Diketodipyrrole		186	70+93+**186**	[4]	U	P
DKP Pro-Ala	$C_8H_{12}N_2O_2$	168	**70**+97+125+168	[5, 6]	F	A
Aminopropanoyl_leucine	$C_9H_{18}N_2O_3$	202	**44**+55+70+86+99+113+**128**+141	NIST	F	A
DKP from Pro-Gly	$C_7H_{10}N_2O_2$	154	83+98+**111**+154	[5, 6]	F	A
DKP Pro-Val	$C_{10}H_{16}N_2O_2$	196	**70**+72+97+125+154	[5, 6]	F	A
DKP Leu-Pro	$C_{11}H_{18}N_2O_2$	210	**70**+86+**154**	NIST	F	A

DKP Pro-Pro	$C_{10}H_{14}N_2O_2$	194	70+96+138+166+**194**	[5, 6]	F	A
Alkylanenitrile C20	$C_{20}H_{39}N$	293	43+**57**+70+83+**97**+**110**+**124**+138+236+250+292	NIST	D	U
Alkylanenitrile C22	$C_{22}H_{43}N$	321	**43**+57+70+83+**97**+110+124+138+236+250+264+278+294	NIST	D	U
***n*-alkenes**						
n-C11:1	$C_{11}H_{22}$	154	56+69+84+…+154	NIST	D	U
n-C13:1	$C_{13}H_{26}$	182	56+69+…+182	NIST	D	U
n-C16:1	$C_{16}H_{32}$	224	56+69+…+224	NIST	D	U
n-C17:1	$C_{17}H_{34}$	238	56+69+…+238	NIST	U	A
n-C18:1	$C_{18}H_{36}$	252	56+69+…+252	NIST	U	A
n-C19:1	$C_{19}H_{38}$	266	56+69+…+266	NIST	U	A
n-C20:1	$C_{20}H_{40}$	280	56+69+…+280	NIST	U	A
n-C21:1	$C_{21}H_{42}$	294	56+69+…+294	NIST	U	U
n-C22:1	$C_{22}H_{44}$	308	56+69+…+308	NIST	U	U
n-C24:1	$C_{24}H_{48}$	336	56+69+…+336	NIST	U	U
***n*-alkanes**						
n-C11:0	$C_{11}H_{24}$	156	57+71+85+…+156	NIST	D	U
n-C12:0	$C_{12}H_{26}$	170	57+71+85+…+170	NIST	D	U
n-C13:0	$C_{13}H_{28}$	184	57+71+85+…+184	NIST	D	U
n-C16:0	$C_{16}H_{34}$	226	57+71+85+…+226	NIST	D	U
n-C17:0	$C_{17}H_{36}$	240	57+71+85+…+240	NIST	F	A
n-C18:0	$C_{18}H_{38}$	254	57+71+85+…+254	NIST	F	A
n-C19:0	$C_{19}H_{40}$	268	57+71+85+…+268	NIST	F	A
n-C20:0	$C_{20}H_{42}$	282	57+71+85+…+282	NIST	F	U
n-C21:0	$C_{21}H_{44}$	296	57+71+85+…+296	NIST	F	P
n-C22:0	$C_{22}H_{46}$	310	57+71+85+…+310	NIST	U	P
n-C23:0	$C_{23}H_{48}$	324	57+71+85+…+324	NIST	F	P
n-C24:0	$C_{24}H_{50}$	338	57+71+85+…+338	NIST	U	P
n-C25:0	$C_{25}H_{52}$	352	57+71+85+…+352	NIST	F	P
n-C27:0	$C_{27}H_{56}$	380	57+71+85+…+380	NIST	F	P
n-C29:0	$C_{29}H_{60}$	408	57+71+85+…+408	NIST	F	P
**Alkan-2-ones**						
Trimethyl-2-pentadecanone	$C_{18}H_{36}O$	268	43+**58**+70+71+…+250+268	NIST	U	U
2-K C21	$C_{21}H_{42}O$	310	43+**58**+59+71+…+310	NIST	U	P
2-K C23	$C_{23}H_{46}O$	338	43+**58**+59+71+…+338	NIST	U	P
2-K C25	$C_{25}H_{50}O$	366	43+**58**+59+71+…+366	NIST	U	P
2-K C27	$C_{27}H_{54}O$	394	43+**58**+59+71+…+394	NIST	U	P
2-K C29	$C_{29}H_{58}O$	422	43+**58**+59+71+…+422	NIST	U	P
2-K C33	$C_{33}H_{66}O$	478	43+**58**+59+71+…+478	NIST	U	P
**Others aliphatics**						
Nonanal	$C_{19}H_{18}O$	142	57+70+…114+124	NIST	U	U
Dodecanal	$C_{12}H_{24}O$	184	57+70+…114+172	NIST	U	U
**Phenols**						
Benzaldehyde	C_7H_6O	106	51+**77+105+106**	NIST	D	U
Phenol	C_6H_6O	94	39+65+66+**94**	NIST	U	U
2- methyl-phenol	C_7H_8O	108	77+79+**107+108**	NIST	U	U
Acetyl-benzene	C_8H_8O	120	51+**77+105**++120	NIST	D	U
3/4- methyl-phenol	C_7H_8O	108	77+79+**107+108**	NIST	U	U
Propyl- phenol	$C_9H_{12}O$	136	77+**107**+136	NIST	U	P
Propenyl-phenol	$C_9H_{10}O$	134	39+77+105+107+**133+134**	NIST	U	P
**Lignins**						
Guaiacol	$C_7H_8O_2$	124	53+81+**109**+124	NIST	U	P
Methyl-guaiacol	$C_8H_{10}O_2$	138	77+95+**123+138**	NIST	U	P

Ethyl-guaiacol	$C_9H_{12}O_2$	152	122+**137**+152	NIST	U	P
4-vinyl-guaiacol	$C_9H_{10}O_2$	150	77+107+135+**150**	NIST	U	P
Vanillin	$C_8H_8O_3$	152	81+109+137+**151+152**	NIST	U	P
4-propenyl-syringol	$C_{11}H_{14}O_3$	197	151+179+**194**	NIST	U	P
**Chlorophyll derived compounds**						
Prist-1-ene	$C_{19}H_{38}$	266	43+**56+57**+69+97+111+126+140+196+222+266	[8]	D	A
Phytene	$C_{20}H_{40}$	280	43+55+**57**+69+**70**+83+97+111+125+126+196+210+280	[8]	F	A
Phytadiene 1	$C_{20}H_{38}$	278	43+57+**68**+82+95+109+123+179+ 208+263+278	[8]	F	A
Phytol	$C_{16}H_{32}O$	297	43+57+69+**71**+81+95+111+**123**+278+280	NIST	F	A
**Steroids**						
Cholesta-3,5-diene	$C_{27}H_{44}$	368	**81+145**+146+213+**247**+255+353+**368**+369	NIST	D	U
Stigmasta-3,5,22-triene	$C_{29}H_{46}$	394	**55**+…+105+133+**145**+159+173+213+228+**255**+282+351+379+**394**+395	NIST	D	U
Stigmastan-3,5-diene	$C_{29}H_{48}$	396	105+121+133+**146+147**+213+255+275+288+**382**+**396**+397	NIST	D	U
Cholesta-3,5-dien-7-one	$C_{27}H_{42}O$	382	159+161+**174**+187+199+227+269+298+367+**382**+383	NIST	U	U
Sitosterol	$C_{29}H_{50}O$	414	**145**+159+213+255+273+303+329+381+396+**414**	NIST	F	U
Stigmastanol	$C_{29}H_{52}O$	416	43+107+147+165+201+**215+233**+383+401+**416**	NIST	F	U
Stigmasta-3,5-dien-7-one	$C_{29}H_{46}O$	410	134+**161+174**+187+253+269+395+**410**+411	NIST	U	U
Stigmast-4-en-3-one	$C_{29}H_{48}O$	412	**124**+149+**229**+271+289+370+397+**412**	NIST	U	U
**Hopanoids**						
22,29,30-trisnorhop-16(17)-ene	$C_{27}H_{44}$	368	69+79+95+…**147+149+191**+231+353+368	[10]	U	B
Urs20en16one	Unknown	424	5+**149+191**+355+384	[2]	U	B
C30 hopene: Hop-17(21)-ene	Unknown	410	135+161+**191**+231+**367**+396+410	[10]	U	B
**(poly)aromatics**						
Toluene	C_7H_8	92	39+65+**91+92**	NIST	U	U
Dimethyl-benzene	C_8H_{10}	106	39+65+77+91+105+**106**	NIST	D	U
Styrene	C_8H_8	104	51+78+103+**104**	NIST	D	U
propenyl-benzene	C_9H_{10}	118	91+115+**117+118**	NIST	D	U
2,3-dihydro-inden-1-one	C_9H_8O	132	77+78+103+**104**+132	NIST	D	U

* Tolu, J., Gerber, L., Boily, J.-F. & Bindler, R. High-throughput characterization of sediment organic matter by pyrolysis–gas chromatography/mass spectrometry and multivariate curve resolution: A promising analytical tool in (paleo)limnology. *Anal. Chim. Acta*. **880,** 93–102 (2015).

[1] Faix et al. (1991) *Holz als Roh- und Werkstoff*, 49: 213-219; [2] Schellenkens et al. (2009) *Organic geochemistry* 40: 678-691; [3] Gupta and Cody (2011), *in N.S. Gupta (ed.), Chitin, Topics in Geobiology* 34, Springer Science+Busines Media ; [4] Schellenkens et al. (2014) *Organic Geochemistry* 77: 32-42; [5] Chen et al., (2009) *Journal of Food Science*, 74: 100-105; [6] Fabbri et al., (2012) *Journal of Analytical and Applied Pyrolysis*, 95: 145-155; [7] Faix et al. (1990) *Holz als Roh- und Werkstoff*, 48: 281-285; [8] Nguyen et al. (2005) *Organic Geochemistry*, 34: 483-497; [9] Gill (1997) Chapter 16. Analytical techniques in organic chemistry; in: Modern analytical Geochemistry; Taylor & Francis, New York (USA), pp. 243-272; [10] Meredith et al. (2008) *Organic Geochemistry*, 39: 1243-1248

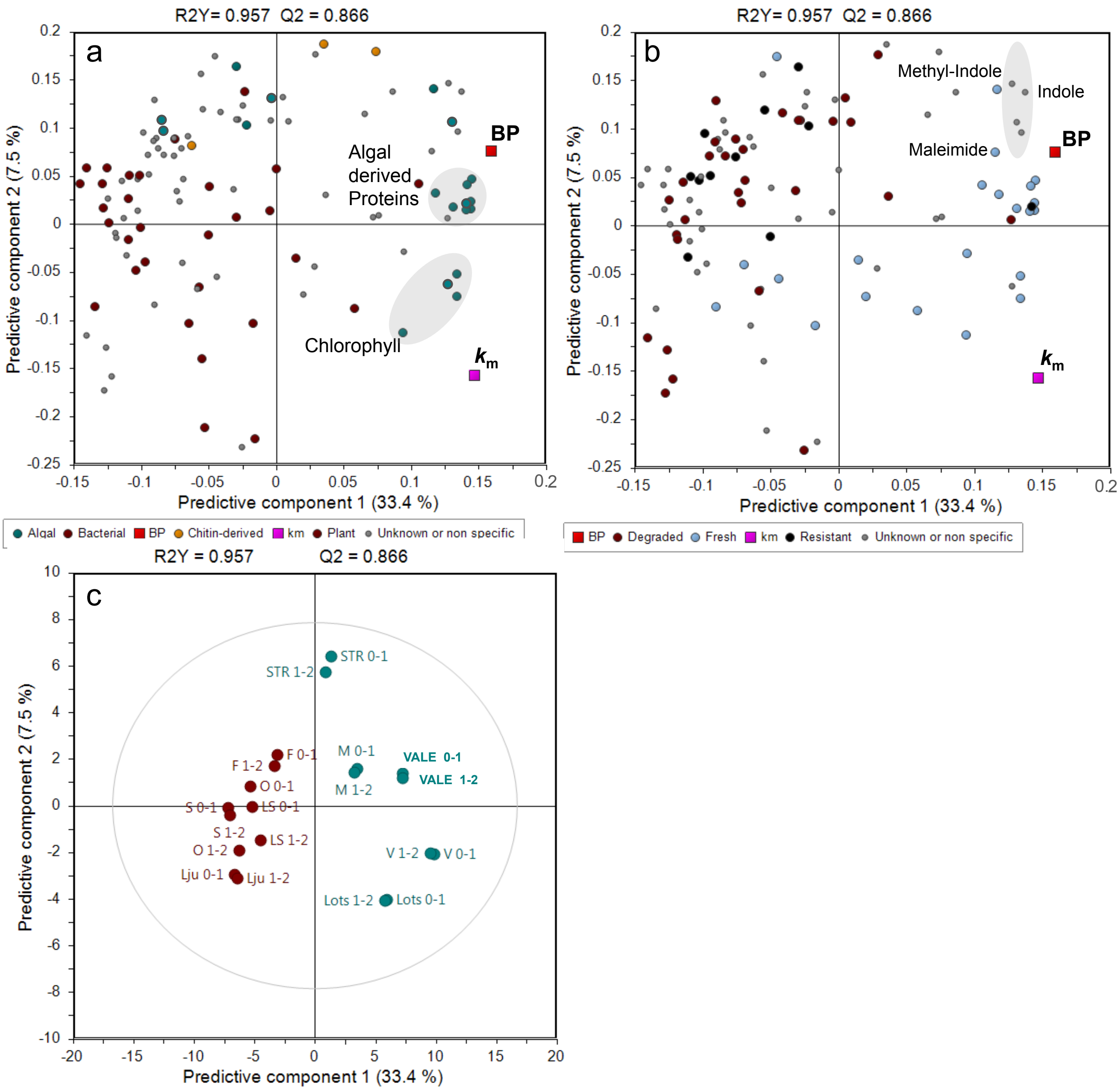

Supplementary Figure 1. Orthogonal Projections to Latent Structures statistical model (OPLS model II) of Hg methylation rate (k_m, pink square) and bacterial production (BP, light red square) as Y variables with the pyrolytic organic compounds as X variables for investigated lakes (0-1 and 1-2 cm). The loadings of OM compounds with predictive capability for BP and k_m, are sorted out into 5 groups according to their origin (a) or into 3 groups according to their degradation status (b). The chart c) presents the scores of lakes dominated by autochthonous (green) or terrigenous (dark red) OM.

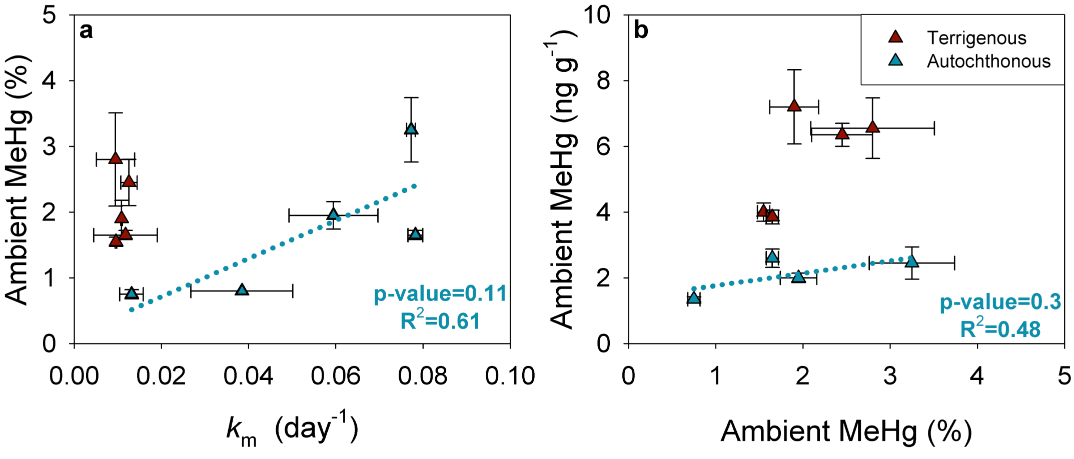

Supplementary Figure 2. Relationship between ambient MeHg percentage and a) Hg methylation rate constants (k_m in day^{-1}) and b) ambient MeHg concentration in lake sediments dominated by terrigenous allochthonous OM (red triangles: Lilla Sångaren, Ljustjärn, Svarttjärn, Fälaren, Oppsvetten) and autochthonous OM (green triangles: Strandsjön, Valloxen, Vallentunasjön, Marnästjärn and Lötsjön).

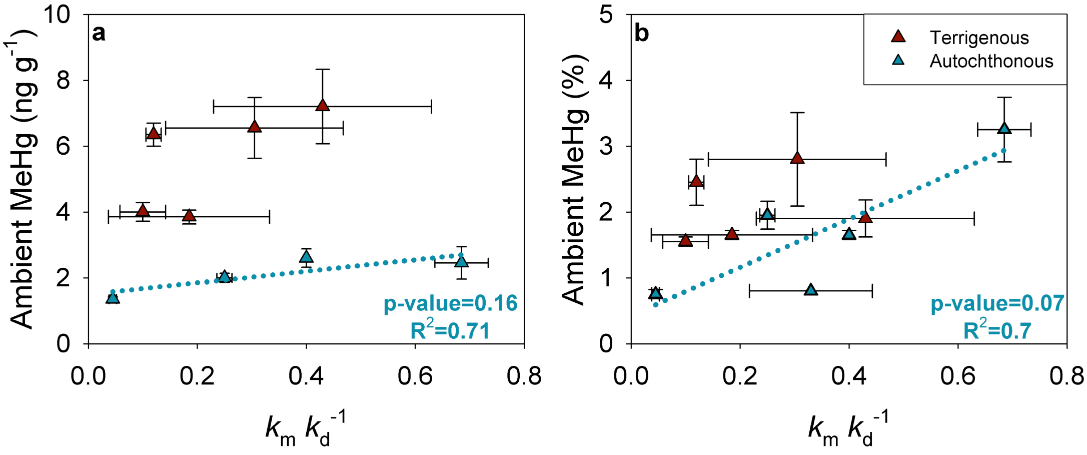

Supplementary Figure 3. Relationships between the net MeHg production (ratio of Hg methylation and MeHg demethylation rate constants, k_m k_d^{-1}) and a) ambient MeHg concentrations or b) ambient MeHg percentage in lake sediments dominated by terrigenous allochthonous OM (red triangles: Lilla Sångaren, Ljustjärn, Svarttjärn, Fälaren, Oppsvetten) and autochthonous OM (green triangles: Strandsjön, Valloxen, Vallentunasjön, Marnästjärn and Lötsjön).
